# Feasibility Study of SBR-Modified Cementitious Mixtures for Use as 3D Additive Construction Materials

**DOI:** 10.3390/polym11081321

**Published:** 2019-08-07

**Authors:** Kwan Kyu Kim, Jaeheum Yeon, Hee Jun Lee, Kyu-Seok Yeon

**Affiliations:** 1 North Gyeonggi Branch, Joongbu Division, Korea Conformity Laboratories, Pocheon, Gyeonggi 11184, Korea; 2 Department of Engineering and Technology, Texas A&M University-Commerce, Commerce, TX 75429, USA; 3 Department of Regional Infrastructure Engineering, Kangwon National University, Chuncheon, Gangwon 24341, Korea

**Keywords:** three-dimensional additive construction, SBR latex, SBR-modified cementitious mixtures, fresh properties, SBR/cement ratio

## Abstract

The primary purpose of this study was to investigate the feasibility of applying polymeric cementitious materials to three-dimensional additive construction (3DAC). Specifically, styrene–butadiene rubber (SBR) latex was employed as an admixture to produce SBR-modified cementitious mixtures, and their fresh properties were experimentally investigated to determine the feasibility of their use in the 3DAC process. The SBR/cement ratio was controlled based on four main materials (i.e., cement, sand, silica fume, and fly ash) in order to determine the optimal fresh properties. The test results revealed that the SBR-modified cementitious mixtures showed excellent flowability, extrudability, buildability, and open time, all of which are required for 3DAC materials. The optimal flow of the SBR-modified cementitious mixtures was 70% ± 1%, which is appropriate for 3DAC applications. According to the experiment results, the SBR-modified cementitious mixtures were sufficiently competitive to serve as a new class of materials for 3D additive construction.

## 1. Introduction

Rapid prototyping technologies, and especially 3D printing, are increasingly being employed in various fields [1]. In particular, such technologies are frequently used in the manufacturing, medical, and foods industries [2,3,4]. Unlike the construction industry, most other major sectors have already established automated systems. While manufacturing automation has rapidly advanced, the propagation of automation in the construction industry has lagged behind other industrial areas. As a result, the construction industry is one of the last to establish automation [5].

Research on the onsite application of three-dimensional additive construction (3DAC) has multiplied. Unlike the conventional method of casting concrete using a formwork, 3DAC integrates multiple areas of advanced knowledge and technology to construct concrete structures based on geometric designs [6]. The 3DAC process is a type of additive manufacturing technology that carries out construction by layering cementitious composites. After this construction method was applied successfully in the construction industry by Pegna [7] in 2015, 3DAC research proliferated around the world. For instance, small buildings and bridges for pedestrians have recently been constructed using 3DAC technology [8,9,10,11].

The process consists of three independent components: a concrete printer, 3D modeling software, and printing material. Studies can be divided into three specialized areas: the mechanical, 3D modeling, and materials fields [12]. The present research addresses the materials specialization. Research is currently being carried out worldwide to develop 3DAC materials applicable for construction projects [13]. These 3DAC materials are frequently produced with Portland cement, fly ash, and silica fume [14,15]. In contrast, in this study, styrene–butadiene rubber (SBR) latex, a water-soluble polymer, was used as an admixture, modifying the cementitious mixtures to improve their workability. The dispersed polymer particles increase the ball-bearing effect and facilitate relative movement between the cement and hydration particles. In addition, the surfactant included in the polymer itself, the latex, acts like a plasticizer to increase the slump value and reduce the amount of water required. In addition, the strength, adhesion, and water resistance are improved considerably [16,17,18,19]. Due to the advantages of adding SBR latex, the fresh properties of the cementitious mixtures studied here all improved, making them appropriate for 3DAC applications. Determining this was the motivation for this study.

However, the addition of the SBR latex must be carefully controlled if the result is to be useful in 3DAC construction [20]. The properties of these fresh 3DAC materials have to be carefully evaluated in terms of flowability (i.e., pumpability), extrudability (i.e., printability), buildability, and open time [14,21,22]; printing cannot commence unless certain property thresholds are achieved. Moreover, these four key properties are not independent of one another, meaning that extrudability, buildability, and open time are all affected by flowability. The present research found that flowability was improved by the ball-bearing effect caused by the addition of SBR latex, and since all four properties are interconnected, it was concluded that the SBR/cement ratio would affect them all. Since the optimal properties of fresh SBR-modified cementitious mixtures using SBR latex and water-soluble polymers as an admixture had yet to be determined, they were experimentally investigated in the present research.

## 2. Materials and Methods

### 2.1. Materials

This study used ordinary Portland cement, silica sand, fly ash, silica fume, superplasticizer, and viscosity modifying agents. Ordinary Portland cement was employed as the main binder, silica sand was used as a fine aggregate, and fly ash was incorporated to improve flowability and inhibit the heat of hydration in the early hydration stage. Silica fume was added to improve strength and reduce segregation in the mixtures. A superplasticizer was included to reduce the water/cement ratio, and a viscosity modifying agent was employed to improve thixotropy. Finally, SBR latex served as a modifier to improve performance by instigating the formation of polymer film.

**Figure 1:**
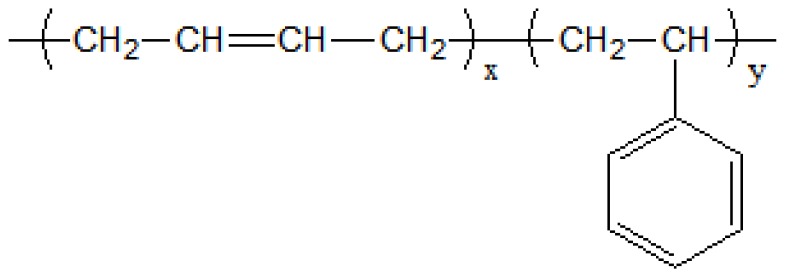
Chemical constitution of styrene–butadiene rubber (SBR) latex.

Their characteristics are outlined below. The features of SBR latex, the key material in the present study, are summarized in Table 1, and the substance’s chemical constitution is illustrated in Figure 1. All of the properties of the materials described in this study were provided by the manufacturers.

- **Ordinary Portland cement** (Type I):
  Density 3.14 (g/cm^3^), specific surface 3630 (cm^2^/g), MgO 2.34 (%), SO_3_ 2.97 (%), LOI 2.76 (%).
- **Silica sand**:
  Particle size 0.08 (mm), apparent density 1.57, SiO_2_ 97.3 (%), Al_2_O_3_ 1.59 (%), TiO_2_ 0.02 (%), Fe_2_O_3_ 0.50 (%).
- **Fly ash**:
  Density 2.22 (g/cm^3^), specific surface 3651 (cm^2^/g), SiO_2_ 51.9 (%), LOI 3.2 (%).
- **Silica fume**:
  Bulk density (undensified) 200–350 (g/cm^3^), specific surface 157,700 (cm^2^/g), SiO_2_ 96.7 (%), LOI 2.39 (%).
- **Superplasticizer** (Policarboxylate)**:**
  Light dark brown liquid, specific gravity 1.05 ± 0.05 (20 °C), pH 5.0 ± 2.0, solid content 20 (%), alkali ≤ 0.01 (%), chloride ≤ 0.01 (%).
- **Viscosity modifying agent** (Organic modified clay):
  White powder, pH (solution) 8.0–10.0, bulk density 430 (kg/m^3^), moisture content ≤12 (%), particle size (0.074 mm) ≥ 95 (%).

### 2.2. Mix Design

The water/cement ratio necessary for the mix design was determined based on the 70% optimal flow for the SBR-modified cementitious mixtures, obtained through an extrusion test. Section 3.1 details the process for determining this optimal flow of 70%. The water/cement ratios that met the 70% optimal flow were determined in relation to the SBR/cement ratios used in this study. These ratios ranged between 0.398 and 0.452, and decreased as the SBR/cement ratio increased. According the ASTM C109/C109M-02: Testing Method for Compressive Strength of Hydraulic Cement Mortar, the standard flow for producing a standard mortar specimen is 110% ± 5% [23], indicating that the optimal flow for the SBR-modified cementitious mixtures was relatively small. Here, the SBR/cement ratio was based on the total solids included in the SBR, and the water/cement ratio was set considering the amount of water included in the SBR latex. The optimal mix proportions based on the above conditions are shown in Table 2.

### 2.3. Printing Setup and Procedure

**Figure 2:**
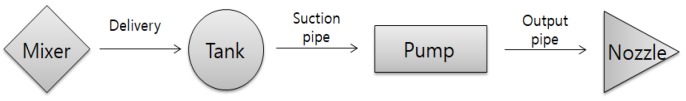
Sequence of the three-dimensional additive construction (3DAC) system [12].

**Figure 3:**
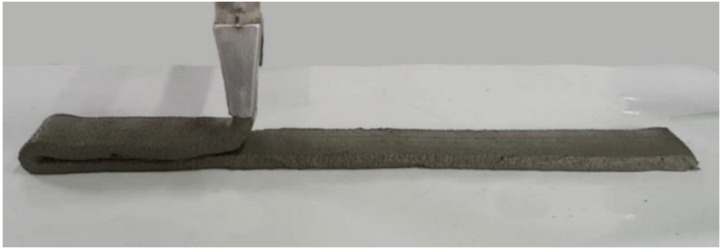
View of 3DAC test.

A custom-made gantry-type extrusion-based 3D printer was used for the 3DAC process. The pumping system was composed of a peristaltic pump (i.e., squeeze pump) and nozzle head. The length of each layer in the printing test was 50 cm and the cross-section of the nozzle where the mortar was output was 5.7 cm (width) × 1.4 cm (height). The sequence of the 3DAC system used in this study is shown in Figure 2. The 3DAC test is illustrated in Figure 3.

### 2.4. Testing Methods

There are no standard test methods for 3DAC. Hence, test methods were identified based on a literature review. The test methods employed are described in Section 2.4.1, Section 2.4.2, Section 2.4.3 and Section 2.4.4.

#### 2.4.1. Flowability

Flowability represents the ease with which the printing material poured into the tank of the printer reaches the printer’s nozzle [14]. This is also referred to as pumpability. The workability test method was selected to test the flowability because there is currently no standard flowability test method for 3DAC. Specifically, the workability test method was selected rather than the Vebe time, compacting factor, or slump test methods because the Vebe time-test method is appropriate only when the workability is very low, while the compacting factor and slump tests are suitable when the workability is medium, and the flow test is preferred when the workability is very high [24].

The flow test method defined in ASTM C1437-15: Standard Test Method for Flow of Hydraulic Cement Mortar was used in the present work, and the results calculated using the following Equation (1) [25]:

(1) $$x=\frac{A}{100}\times 100$$

where *x* is the flow (%) and *A* is the average of the four measurements of the diameter after the flow test (mm).

#### 2.4.2. Extrudability

Extrudability represents the ease with which the printing material is continuously extruded from the nozzle, also referred to as printability. Extrudability is the extrusion rate (in cm/min or cm/sec) of the cementitious mixture passing through the nozzle.

In this study, to assess extrudability, the extrusion time was measured until 10 concrete layers were stacked vertically. The horizontal length of each concrete layer was 50 cm. Extrudability was calculated using Equation (2):

(2) E=L/T

in which *E* is the extrudability (cm/min), *L* is the total extrusion length (cm), and *T* is the time duration (min) until the end of the extrusion.

#### 2.4.3. Buildability

Buildability is the capacity of the layers of a cementitious mixture extruded through a printer nozzle to stably sustain themselves when stacked vertically. A buildability test assesses the number of stacked layers, as well as their height and vertical displacement, according to the time duration after layering has ceased.

In this study, the stacked height and decrease in height after layering were both measured. The same stacking condition (i.e., 10 stacked layers, each 50 cm in length) was used as the extrudability measurement. Here, 10 layers were selected because it was determined in numerous previous stacking tests that this number of layers would remain stable and not collapse.

#### 2.4.4. Open Time

Open time refers to a time period beginning when a sample of concrete is first able to consistently maintain itself and ending the moment the concrete cannot be used. In 3DAC, open time refers to the time duration beginning with extrusion and ending with when extrusion is no longer possible due to degraded flowability. Open time represents the relationship between this time duration and any deterioration in workability. Using the flow test method, open time was determined based on the measurement data related to flowability changes during a specific time duration.

## 3. Results and Discussion

### 3.1. Determination of Optimal Flow

In order to carry out 3DAC work in a stable manner, consistent flowability must be maintained. This is especially important in concrete printing, since the cementitious mixtures must extrude smoothly from the tank of the concrete printer to and through its nozzle. The conditions needed for uniform and stable layering of the extruded mixtures must also be determined. Flow serves as the basis for these conditions. Typically, rotational or vibrational rheometry equipment is also used to assess plastic viscosity, yield stress, and thixotropy. However, this method is not appropriate for 3DAC materials that exhibit high yield stress and viscosity [14,26,27].

**Figure 4:**
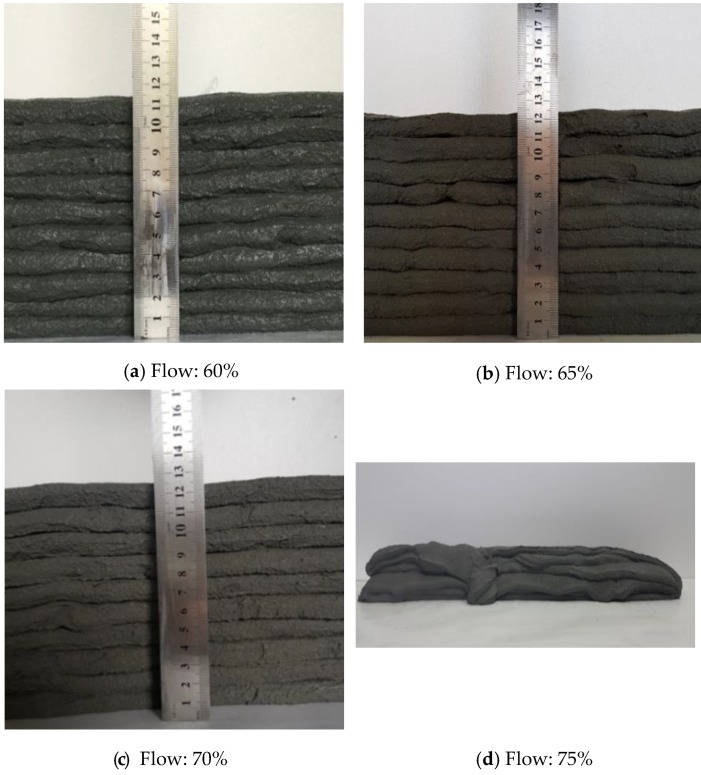
Test results of flow determination (SBR/cement ratio: 0.15).

Taking all of these aspects into consideration, in this study flow was measured using the method defined by ASTM C1437-15 [25]. The flow was varied by intervals of 5%, beginning with 55% and ending with 75%; printing tests were also conducted. Here, the SBR/cement ratio was set to 0.15. The results for the 60%, 65%, and 70% flows were 11.5 cm, 11.3 cm, and 12.5 cm, respectively (see Figure 4). Extrusion was not possible for the 55% flow due to low flowability, but was possible for the 75% flow. However, in the latter case, layering was not possible because of the high flowability. The 70% flow resulted in the highest extrudability and buildability, so the 70% flow was determined to be optimal.

There have been several earlier studies on flow. Malaeb et al. [21] conducted a slump flow test following the parameters outlined in ASTM C1611/C1611M-14: Standard Test Method for Slump Flow of Self-Consolidating Concrete. Their research examined cementitious mixtures produced using cement, sand, aggregate, and plasticizer. The authors determined that there was a problem with extrudability when the slump flow was lower than 1.0 cm/s, and buildability was negatively affected when the slump flow was greater than 1.2 cm/s. There have also been tests using the method outlined in ASTM C1437: Standard Test Method for Flow of Hydraulic Cement Mortar. Kazmian et al. [5] found that the flows of cementitious mixtures produced using silica fume, fiber, and nanoclay with Portland cement as the base were in the range of 113% to 119%. Marchment et al. [28] determined that the flow was 82% for ordinary Portland cement, coarse sand, and fine sand when the W/C ratio was 0.38. Thus, the appropriate flow varied depending on certain factors such as the materials used and test method employed.

### 3.2. Flowability

Flowability is an important element and critical for ensuring 3DAC workability. The present study analyzed and measured flow variation according to the time duration after mixing. For an optimal flow of 70%, measurements were made at 30-min intervals for the first hour, followed by 20-min intervals for the next 80 min. Measurements were only made for a total of 140 min because extrusion became impossible at that point for an SBR/cement ratio of 0.2.

**Figure 5:**
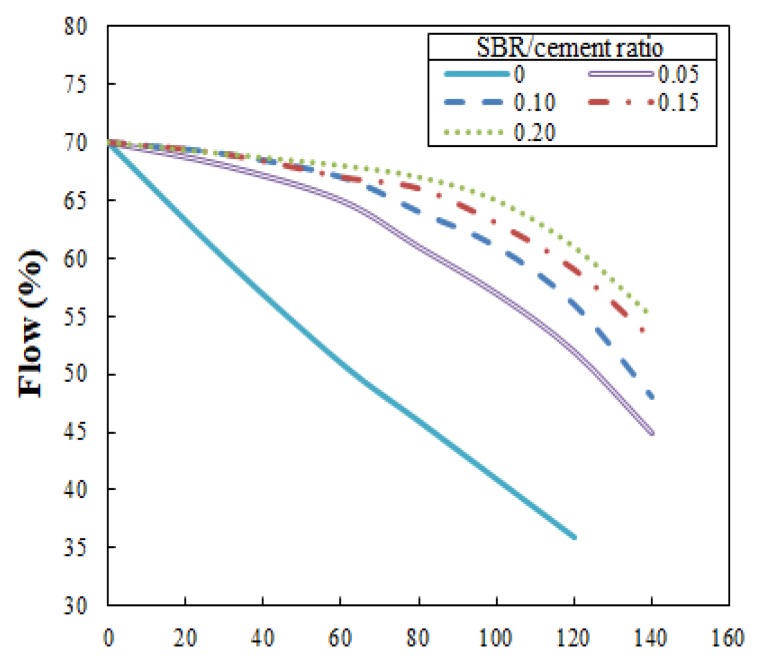
Elapsed time versus flow.

**Figure 6:**
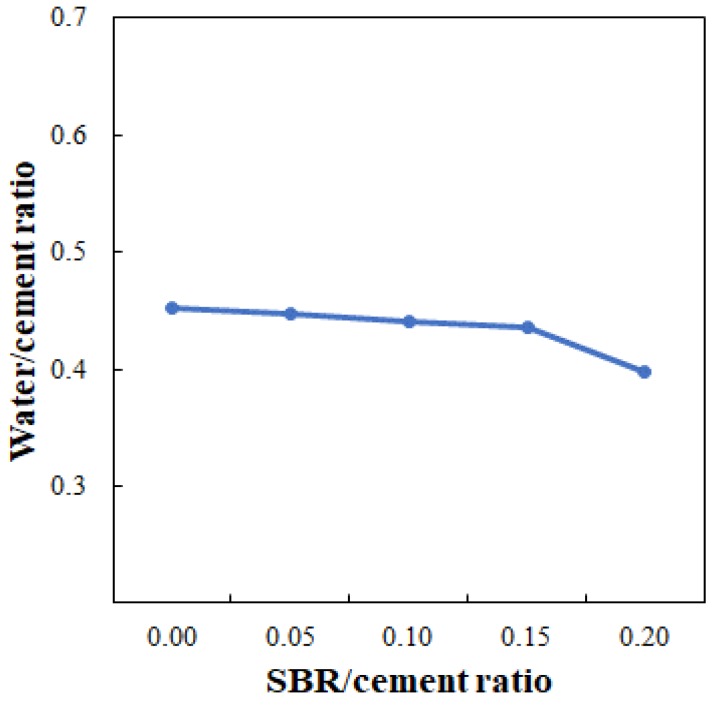
Water/cement ratios required to determine the optimal flow of 70%.

The test results are illustrated in Figure 5, which shows that the flow loss decreased as the SBR/cement ratio increased. The water/cement ratios at which the flow reached 70% (see Figure 6) were 0.452, 0.448, 0.441, 0.436, and 0.392 for SBR/cement ratios of 0, 0.05, 0.10, 0.15, and 0.20, respectively. From this result, it was determined that securing the necessary flowability in the cementitious mixtures would not be problematic, even when the SBR/cement ratio increased and water/cement ratio decreased.

### 3.3. Extrudability

When extrusion of the cementitious mixtures is not possible, there is no need to consider other properties; they are not important. Extrusion becomes difficult when the cementitious mixture is not fluid. Stacking is complicated if extrusion is not smooth, and the printed structure will be unstable if stacking is not carried out to quality. Since extrudability relies on flowability, it is affected by the types, properties, amounts, and moisture content levels of the materials that comprise the cementitious mixture, along with the presence of additives, type of transfer system, and amount of transfer time.

**Figure 7:**
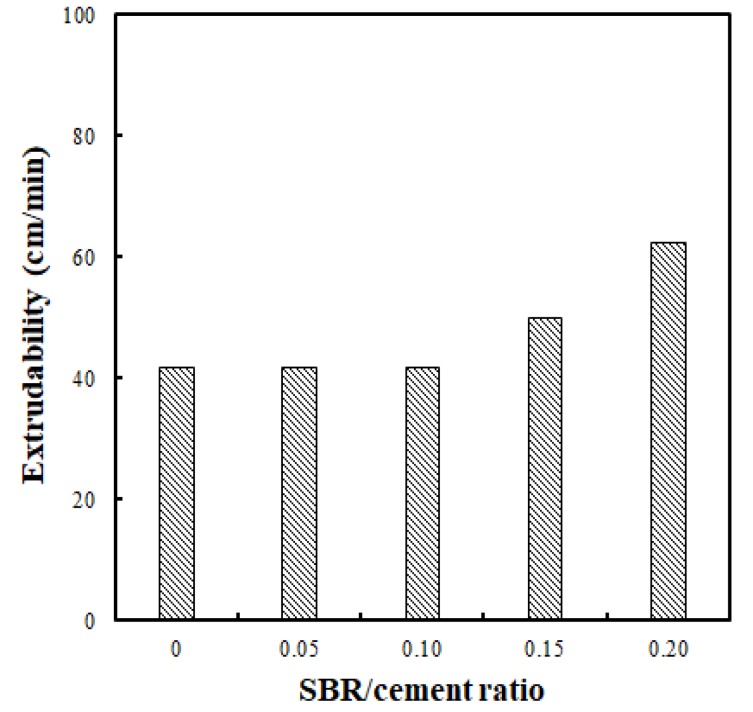
Extrudability comparison for different SBR/cement ratios.

Extrudability was tested in this study by continuously printing a total length of 500 cm, at a height of 10 layers and length of 50 cm. The test conditions used an optimal flow of 70% and feed rate of 1.2 rpm for the peristaltic pump. The test results shown in Figure 7 indicate that the extrudabilities were 41.7 cm/min, 41.7 cm/min, 41.7 cm/min, 50.0 cm/min, and 62.5 cm/min for the SBR/cement ratios of 0, 0.05, 0.10, 0.15, and 0.20, respectively. From the results, it was determined that the operating speeds were similar for SBR/cement ratios ranging from 0 to 0.10, but the speed increased significantly above an SBR/cement ratio of 0.15. This improvement in extrudability, even when the flow and operation speed were kept constant, was the result of the ball-bearing effect produced by the polymer particles during stirring of the SBR latex into an emulsion form [16]. However, extrudability could vary depending on the shape and size of the structure being printed. The natural environment (e.g., the surrounding temperature and humidity), as well as the type of printing equipment (including the pump), can both affect extrudability.

A previous study [27] determined the optimum operating speed to be between 30 mm/s and 35 mm/s when a rectangular nozzle (40 mm × 10 mm) is used, based on a gantry-type concrete printer, and 50 mm/s to 66 mm/s when a 9 mm circular nozzle is employed. It was also reported that the operating speed exceeded 300 mm/s when a robotic arm-type of concrete printer was selected.

### 3.4. Buildability

Buildability refers to the number of layers that can sustain themselves on their own after they have been stacked. Therefore, the test for buildability is related to the number of layers that can be achieved. Higher buildability indicates a greater number of cementitious mixture layers that can be stacked before excessive deformation and collapse results [21]. Ultimately, buildability is related to the stiffness of the extruded material in the early stages of printing. Suitable buildability is a basic requirement for the cementitious mixtures used in 3DAC [29]. However, 3DAC also requires the formation of uniform layers through sequential layering of the cementitious mixture. Because there is a high possibility of cold joints being formed between layers (as compared to the traditional concrete casting method), pausing the layering process is not ideal [30].

**Figure 8:**
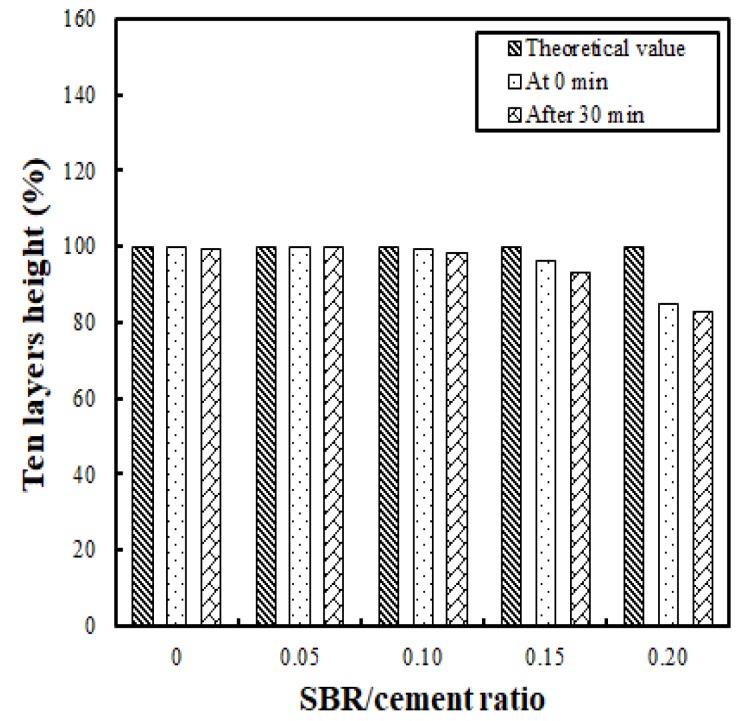
Comparison of buildability levels for different SBR/cement ratios.

In this study, buildability was evaluated by measuring the height immediately after stacking 10 layers 50 cm in length, and then measuring the height after 30 min. The height was measured after 30 min because preliminary testing revealed that there was barely any height change after this point. The results of the buildability test are shown in Figure 8. It was found that the heights immediately after stacking were 12.8 cm, 13.1 cm, 13.2 cm, 12.5 cm, and 11.4 cm for the SBR/cement ratios of 0, 0.05, 0.10, 0.15, and 0.20, respectively.

When the height measurements were compared with the theoretical value (the one-layer height × 10 layers), the difference was very small—less than or equal to 0.1 cm for SBR/cement ratios ranging from 0 to 0.10. However, the change in SBR/cement ratio of 0.15 to 0.20 showed a drastic increase—between 0.5 and 2.0 cm. Comparison of this result with the height after 30 min showed that the difference was minimal—at less than or equal to 0.1 cm for SBR/cement ratios of 0 to 0.10. The difference increased to 0.3 to 0.4 cm for the SBR/cement ratios of 0.15 to 0.20.

**Figure 9:**
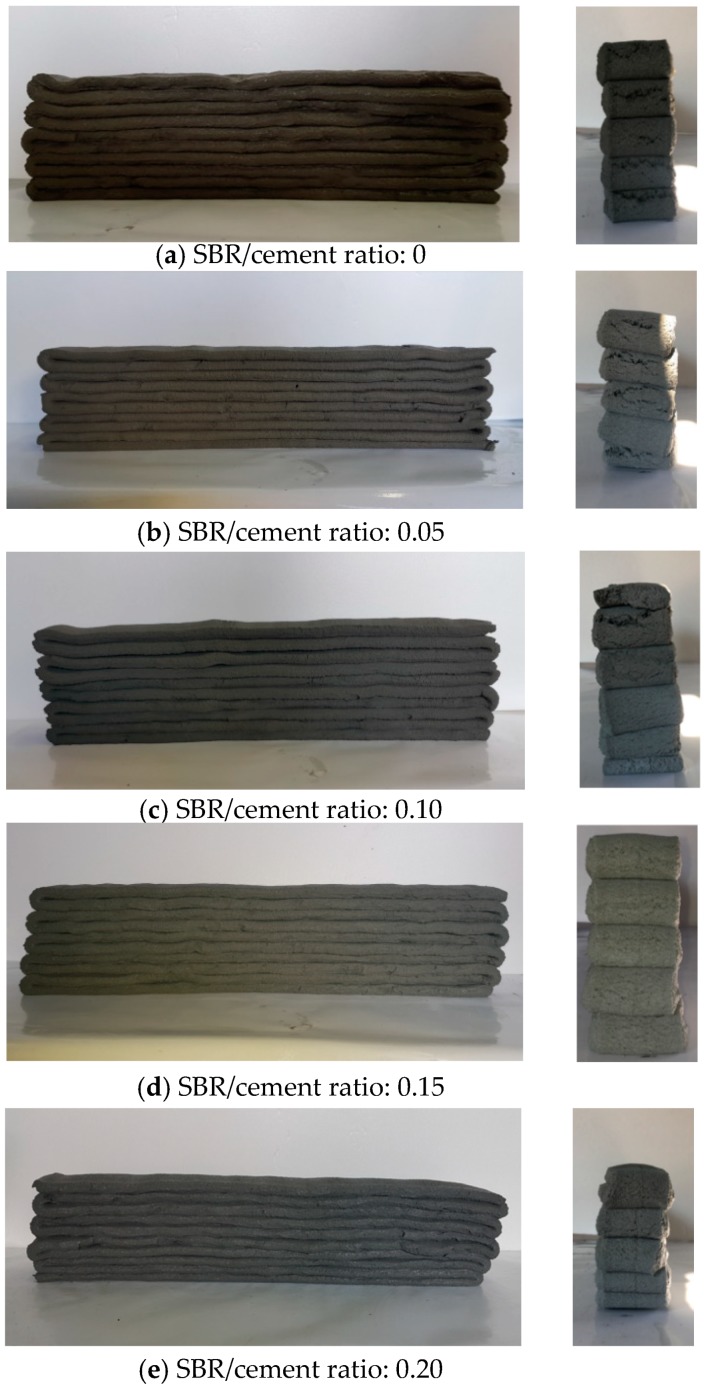
Test results for buildability (flow: 70%).

It is interesting to note that cracking occurred at the ends of the printed layers (the point where the nozzle changed direction) when the SBR/cement ratio was less than or equal to 0.10, even though the flow of 70% was kept constant (see Figure 9). Cracking did not occur for SBR/cement ratios of 0.15 and 0.20. Accordingly, it was determined that the SBR/cement ratios that did not result in cracking and had relatively little stacking height reduction ranged between 0.10 and 0.15. The additive effect of SBR is thought to be related to Ohama (1995), who showed that although the additive effect of SBR is high compared to the lack of a flow change in polymer-modified cementitious mixtures, the resistance to bleeding and segregation is significant [31].

### 3.5. Open Time

Open time is a very important factor when considering the 3DAC properties. Cementitious mixtures prepared for printing are not cast all at once, as is the case in the traditional cast-in-place concrete method. Hence, some properties can change due to the time difference between the printing’s beginning and end. For example, extrusion becomes increasingly difficult as flowability degrades with time; the time duration ending at the point at which this phenomenon takes place is called open time.

Open time is the time during which the flowability of cementitious mixtures can be maintained. It is an important property necessary to establishing 3DAC operating times and essential to determining variations in flowability that occur over time [21]. Generally, flow decreases as time passes. For instance, Marchment et al. [28] used flow tests conducted according to ASTM C1437 for mortar produced using cement, sand, water, and additives, showing that the flow decreased from 66% (measured after 3 min) to 51% (measured after 15 min).

In the present study, to obtain the open times for SBR-modified cementitious mixtures, the optimal flow of 70% was used; measurements were made at 30-min intervals for the first hour, followed by 20-min intervals for the next 80 min. Measurements were made for a total of 140 min because after that extrusion was impossible when the SBR/cement ratio was 0.20 (as mentioned in Section 3.2). The open time for 3DAC is conceptually similar to the notion of working life used for paints and varnishes and initial and final setting times used for cements, though the test methods for these time concepts cannot be employed for this type of material.

**Figure 10:**
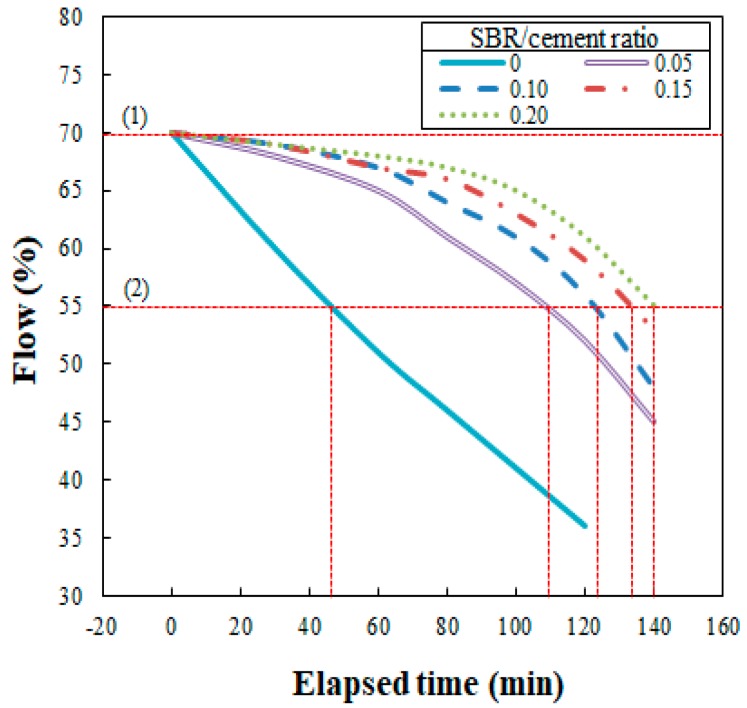
Delay time versus flow, used in finding the open time.

**Figure 11:**
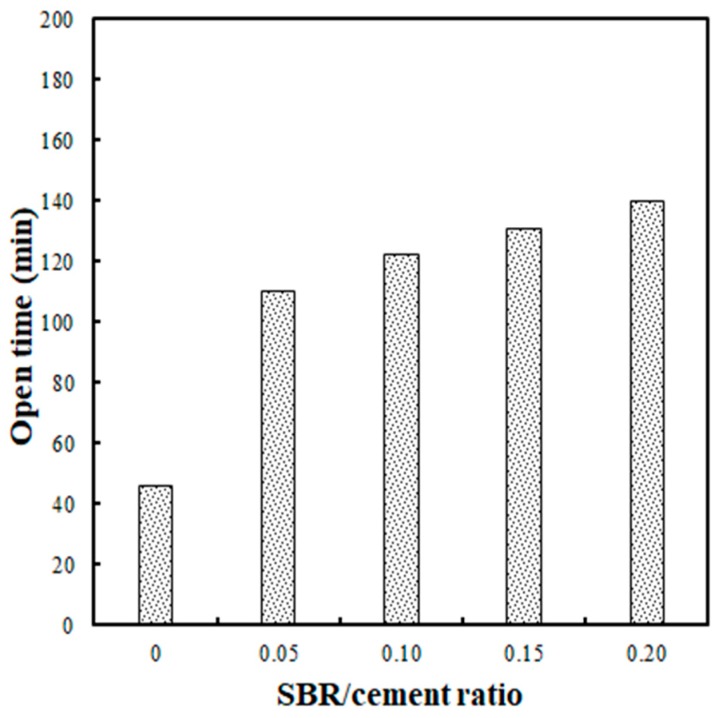
Comparison of open times for different SBR/cement ratios.

The flow test results for 140 min after the extrusion of the SBR-modified cementitious mixtures are shown in Figure 10. In Figure 10, (1) indicates the test’s starting point when the flow was 70%, and (2) marks the open time or time duration until a flow of 55% was reached, after which extrusion was not possible. As shown in Figure 11, the open times were 46 min, 110 min, 122 min, 131 min, and 140 min for the SBR/cement ratios of 0, 0.05, 0.10, 0.15, and 0.20, respectively. An important aspect of these results is that the open time increased as the SBR/cement ratio was increased. This is thought to be due to the delay in the initial setting as the polymer film formed, suppressing the initial hydration reaction of the cement. Similar studies have found that the setting time of SBR-modified cementitious mixtures is delayed compared to when only ordinary Portland cement is used [16]. Ultimately, when engaging in 3DAC projects, increasing the SBR/cement ratio is advantageous to improving the open time.

## 4. Conclusions

This study was carried out as to evaluate the feasibility of using SBR-modified cementitious mixtures in 3DAC applications. The properties of fresh SBR-modified cementitious mixtures were experimentally investigated using various SBR/cement ratios. The following conclusions were drawn from the results.

1. Extrusion became impossible when the flow was lower than 55%, and layering was not possible when the flow was higher than 75%. The optimal flow to facilitate extrusion and layering was 70%, but a flow of 70% ± 1% was ideal, considering deviation.
2. The flowability test results showed that the flow loss decreased as the SBR/cement ratio increased. Also, the water/cement ratio decreased, which was advantageous for the flowability of the cementitious mixtures.
3. The extrudability increased by 20% and 50% for SBR/cement ratios of 0.15 and 0.20, respectively, compared to the SBR/cement ratios of 0 to 0.10. The extrudability improved due to an increase in the ball-bearing effect of the polymer particles as the SBR/cement ratio increased.
4. The buildability test revealed that the reduction in stacked height was relatively small, 0.10 to 0.15 at an SBR/cement ratio of 70% flow, at which point cracking did not occur at the transition of the nozzle direction.
5. The open time increased as the SBR/cement ratio increased. This trend was the result of an initial setting delay, due to suppression of the hydration reaction of the cements by the polymer film that formed within the cementitious mixtures.

The results show that the fresh SBR-modified cementitious mixtures improved in terms of four properties required for 3DAC, signifying that the mixtures are sufficient to be competitive as new concrete printing materials.

## 5. Patents

This research is part of the patent application (Application No. 10-2019-0047891) applied for at the Korean Intellectual Property Office.

## Figures and Tables

**Table 1:** Properties of SBR latex.

Total Solids (%)	pH	Viscosity (mPa·s)	Surface Tension (Dynes/cm)	Specific Gravity (20 °C)	Minimum Film Forming Temperature (℃)
47–50	9.9–10.5	40	30–35	1.01 ± 0.01	<4

**Table 2:** Mixture proportions of SBR-modified cementitious mixtures (unit: kg/m^3^).

SBR	Cement	Water	Silica Sand	Fly Ash	Silica Fume	Superplasticizer	Viscosity Modifying Agent
0	642	289	1377	184	92	6	0.3
32	638	271	1368	182	91	6	0.3
63	635	254	1360	181	91	6	0.3
95	631	236	1351	180	90	6	0.3
125	627	219	1343	179	90	6	0.3
